# Supplementary material for: Determinants of secondary prophylaxis for childhood rheumatic heart disease in Ethiopia: A qualitative study of children and caregivers
Source: PLoS One. 2026 May 26;21(5):e0349776. doi: 10.1371/journal.pone.0349776 (PMC13210385; doi:10.1371/journal.pone.0349776)
Supplement: S1 Text — (DOCX) [file pone.0349776.s001.docx]

**Supplementary 1 Text: Focus group discussion guide**

1. **Experience with Diagnosis and Treatment:**

- Can you share your experience of being diagnosed with ARF/RHD and receiving prophylactic treatment for rheumatic fever or heart disease?

1. **Perceptions of BPG Secondary Prophylaxis:**

- How do you view the goals and services related to BPG secondary prophylaxis? What challenges have you encountered with adherence?

1. **Factors Impacting Prophylaxis delivery services:**

- What factors influence the delivery of BPG prophylaxis in healthcare settings? Consider:
- Can you tell us about things that make it easy or hard for you to get your penicillin injections?”
- Follow-up probes (used flexibly by facilitators as needed):
- “What is it like when you go to the clinic for your injection?”
- “How do nurses or health workers treat you when you go for your injection?”
- “Is there anything about the injection that makes you afraid or uncomfortable?”
- “Who helps you remember or come for your injection?”

1. **Alternative Medications:**

- Have you used any other medications for secondary prophylaxis besides BPG?
  - If yes, what medications, for how long, and what influenced your choice to switch or start?
  - How do these compare to BPG in terms of effectiveness and preference?

1. **Suggestions for Service Improvement:**

- What improvements would you suggest for RHD services? If you could speak with the Chief of the Federal Ministry of Health or a WHO representative, what key points would you raise?

1. **Closing Questions:**

- What do you think is the most important issue from today’s discussion?
- Are there any final thoughts or aspects we missed that you’d like to share?
