# Supplementary material for: Determinants of secondary prophylaxis for childhood rheumatic heart disease in Ethiopia: A qualitative study of children and caregivers
Source: PLoS One. 2026 May 26;21(5):e0349776. doi: 10.1371/journal.pone.0349776 (PMC13210385; doi:10.1371/journal.pone.0349776)
Supplement: S2 Table — (DOCX) [file pone.0349776.s003.docx]

Supplementary 2 Table: The Capability, Opportunity, Motivation and Behaviour (COM-B) model’s domains and their definitions

| COM-B model domains | Definitions |
| --- | --- |
| Capability (psychological and physical | Individual person’s psychological and physical capacity to engage and perform the desired activity or behaviour. This includes having the required necessary knowledge and skills. |
| Opportunity (social and physical) | All external factors in person’s environment or circumstances that encourage or discourage the desired behaviour. This includes time, resource, and norms of practice) |
| Motivation (reflective and autonomic) | A coherent set of brain process that determines person’s displayed personal qualities in a social and work setting. This includes habitual processes, emotional responding, analytical decision making and professional confidence. |

Adopted from Michie S, van Stralen MM, West R. The behaviour change wheel: a new method for characterising and designing behaviour change interventions. Implement Sci 2011; 6:42–53.
