# Supplementary material for: Determinants of secondary prophylaxis for childhood rheumatic heart disease in Ethiopia: A qualitative study of children and caregivers
Source: PLoS One. 2026 May 26;21(5):e0349776. doi: 10.1371/journal.pone.0349776 (PMC13210385; doi:10.1371/journal.pone.0349776)
Supplement: S1 Fig — (DOCX) [file pone.0349776.s004.docx]

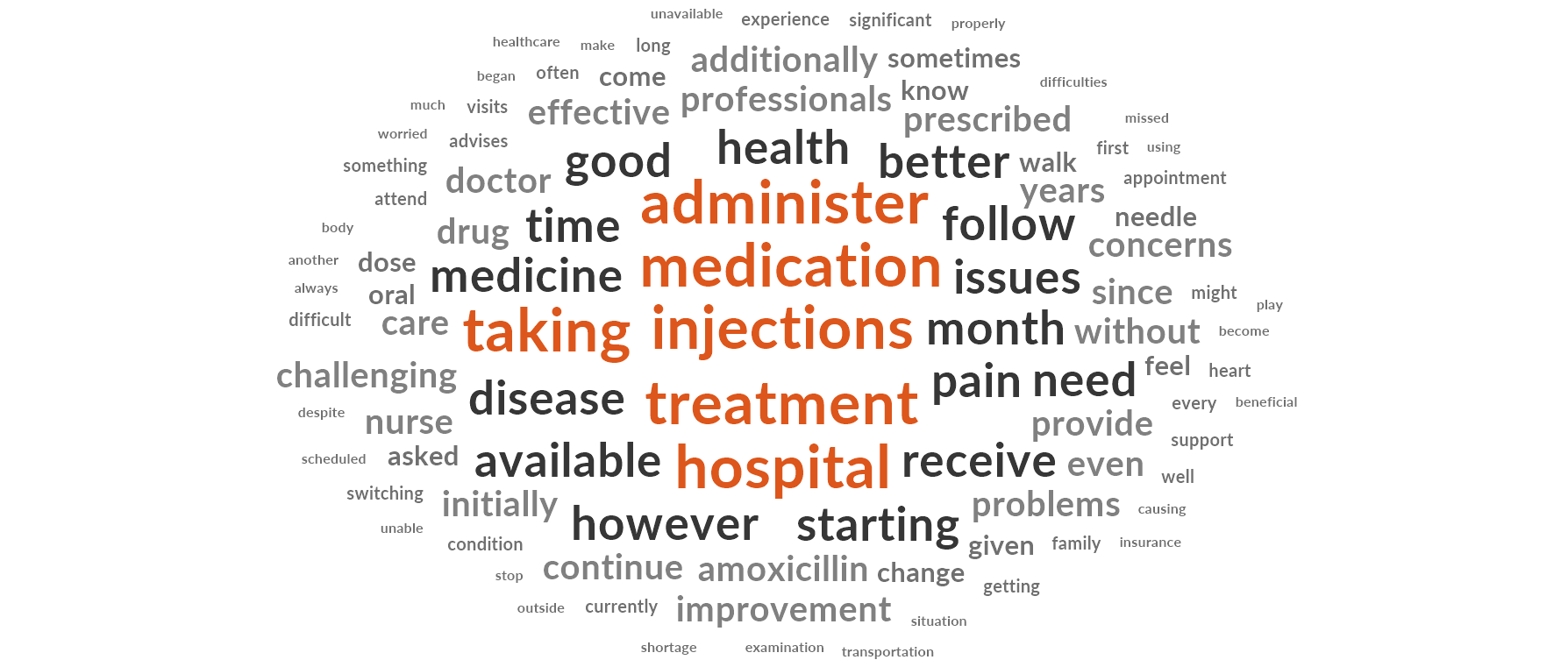


Supplementary 1 Figure: Word cloud of participants transcript (100 most frequent with a minimum length of 4)
