# Supplementary material for: Determinants of secondary prophylaxis for childhood rheumatic heart disease in Ethiopia: A qualitative study of children and caregivers
Source: PLoS One. 2026 May 26;21(5):e0349776. doi: 10.1371/journal.pone.0349776 (PMC13210385; doi:10.1371/journal.pone.0349776)
Supplement: Attachment — Submitted filename: Response to reviewers.docx [file pone.0349776.s007.docx]

18^th^ February 2026

Dear Editor,

Thank you for the opportunity to submit a revised version of the article entitled “Determinants of secondary prophylaxis for childhood rheumatic heart disease in Ethiopia: a qualitative study of children and caregivers". We are grateful to the reviewers for the opportunity to improve this piece of work and have carefully addressed each of their comments and revised the manuscript accordingly. In particular, we have clarified participant inclusion criteria, expanded reporting of clinical characteristics, strengthened the Discussion with relevant comparative and contextual literature, and improved methodological transparency where suggested.

A detailed, point-by-point response to each comment is provided below, with corresponding revisions clearly indicated in the manuscript.

1. (R1): The title: suggest to change to Determinants of " secondary prophylaxis for RHD" delivery as the study contains 30% of patients not receiving BPG.

We agree and have revised ((Page 1, Line 1-2).

2. (R1): Introduction: need to emphasize the differences between this study and the similar one that was conducted in Uganda

We thank the reviewer for this important comment. We have revised the Introduction to more clearly distinguish the present study from the similar qualitative study conducted in Uganda, and have added an encompassing reference to the social, economic, and health-system barriers and enablers to RHD care assessed across these studies (Page 4, Line 99-102).

3. (R1): The patient information regarding clinical status is important (stage of disease, heart failure), also need to include the reason for changing from injection to oral as that affects many other factors.

We agree that clinical status is important for interpreting participants’ experiences and factors influencing secondary prophylaxis delivery. Although eligibility included ARF and RHD patients on secondary prophylaxis for ≥6 months, all enrolled participants had symptomatic RHD—likely reflecting delayed presentation and diagnosis in resource-limited settings. Of the cohort, 47% had symptomatic RHD without heart failure and 53% had RHD with heart failure; none had complicated RHD. We have clarified these clinical characteristics in the Results section. In addition, we have clearly described the reasons for switching from injectable benzathine penicillin G to oral prophylaxis and these considerations have now been addressed in Results, and Discussion sections (Page 7, Line 225-233).

4. (R1): Discussion: more in-depth discussion about BPG side effects, they include nonallergic fatalities and the emphasis on allergy needs to be less, rather, discuss this nonallergic reactions and need for training the health care personnel following WHO latest guidelines 2024 and reports from the American Heart Association

We thank the reviewer for this important clarification. We agree that an overemphasis on allergic reactions may obscure the broader safety profile of BPG. We have revised the Discussion to (i) de-emphasise allergy as the sole safety concern, (ii) explicitly discuss rare but serious non-allergic adverse events (including cardiovascular collapse and injection-related fatalities), and (iii) emphasise the need for healthcare worker training, preparedness, and adherence to current WHO (2024) guidance and American Heart Association recommendations. These revisions better contextualise provider fear, clarify that most severe events are non-allergic, and highlight actionable system-level solutions rather than avoidance of BPG use (Page 15-16, Line 477-489).

5. (R1): Comparing the results of this study with the similar one done in Uganda is needed emphasizing methodologic differences and outcomes

We thank the reviewer for this valuable suggestion. We agree that clearer comparison with the similar qualitative study in Uganda strengthens interpretation of our findings and have expanded the Discussion accordingly. The Ugandan study used in-depth interviews across a wide age range and examined the broader lived experience of RHD and multilevel barriers and enablers to care. In contrast, our focus groups with children with symptomatic RHD and their caregivers enabled exploration of shared and divergent child–caregiver perspectives and caregiver-mediated decision-making, with a specific focus on BPG delivery (including centre-level variation, provider refusal, practical challenges, and switching to oral prophylaxis). While both studies identified common system constraints, our findings extend the Ugandan work by foregrounding child distress, caregiver burden, and provider risk perception as key influences on BPG delivery in Ethiopia (Page 14, Line 428-441).

6. (R1): May include in discussion the research on subcutaneous BPG. Kado JH, Salman S, Henderson R, Hand R, Wyber R, Page-Sharp M, Batty K, Carapetis J, Manning L. Subcutaneous administration of benzathine benzylpenicillin G has favourable pharmacokinetic characteristics for the prevention of rheumatic heart disease compared with intramuscular injection: a randomized, crossover, population pharmacokinetic study in healthy adult volunteers. J Antimicrob Chemother. 2020 Oct 1;75(10):2951-2959. doi: 10.1093/jac/dkaa282. PMID: 32696033.

As suggested, we have incorporated the above reference and expanded the Discussion to include evidence on subcutaneous administration of benzathine penicillin G (BPG), highlighting its favourable pharmacokinetic characteristics and potential relevance for addressing injection-related barriers identified in this study (Page 16, Line 491-499).

7. (R2): Methods, Participant inclusion: Child participants in the study were defined as "C-RHD <18 years of age who were receiving specialised care and treatment for ARF/RHD for at least 6 months..." (Methods paragraph 4, page 4). The term "ARF/RHD" here creates ambiguity here: did all the children have confirmed RHD but some had a history of diagnosed ARF, or does it mean that children could be included if they had either ARF or RHD (or both), therefore some might have had ARF alone and no RHD? If the latter is true, the descriptor "C-RHD" (children with RHD) seems inaccurate, and the lived experience of those who were solely diagnosed with ARF is likely different from those diagnosed with RHD. I suggest further clarification, for example that all child participants had been diagnosed with RHD, if this is correct.

Thank you for this important observation. We agree that the term “ARF/RHD” in the Methods section could create ambiguity. Although the eligibility criteria permitted enrolment of children with ARF or RHD receiving secondary prophylaxis for at least six months, all participants ultimately enrolled and interviewed had a confirmed diagnosis of symptomatic RHD at the time of recruitment. No children with isolated ARF without established RHD were represented in the final study sample. This reflects the clinical context of the study sites, where children commonly present with established RHD and isolated ARF is less frequently managed within specialised services. We have revised the Results section to explicitly clarify this point and ensure that the descriptor “C-RHD” accurately reflects the study population (Page 7, Line 225-228).

8. (R2): Researcher positionality and language. Positionality is only described in terms of gender (paragraph 2, page 5), and ethnicity, nationality and language fluency are not mentioned. The paper does not clearly state the language used in the focus group discussions, or whether multiple languages were used. Transparency in this area is important to allow readers to consider possible bias, translation effects and dynamics between participants and facilitator/researchers. I suggest that the authors briefly expand on the information provided about the focus group methods, to include ethnicity of facilitators, language(s) spoken during focus groups, and whether researchers shared language(s) with participants or whether translators were used during the focus groups.

Thank you for this thoughtful comment. We agree that transparency regarding researcher positionality and language is important for interpreting potential sources of bias, translation effects, and group dynamics in qualitative research, and have incorporated this into the manuscript. Ethnicity data were not collected from participants or facilitators due to cultural sensitivity and the prevailing sociopolitical context, where explicit identification or documentation of ethnicity was considered inappropriate. All participants were Ethiopian nationals. Focus group discussions were conducted primarily in Amharic, except at Dire Dawa where discussions were conducted in Afan Oromo. Facilitators were fluent in the language used at each site and no external interpreters were involved. Audio recordings were later translated into English for analysis by a bilingual member of the research team. We also note these contextual constraints in the Discussion (Page 6, Line 187-196).

9. Results, Participants' ethnicity and language: Sociodemographic characteristics provided on children with RHD participating in the study (Results paragraph 1, page 5, and Table 1, pages 5 and 6) do not include ethnicity or primary languages spoken. It would be helpful to include these data if available to help readers' understanding of the diversity of participants.

We thank the reviewer for this important comment. Participants’ ethnicity and primary language were not collected in the sociodemographic survey. This was a deliberate decision based on cultural sensitivity and contextual considerations at the time of data collection, including heightened ethnic-based tensions and political sensitivities, such that asking participants to identify ethnicity was not considered appropriate. All child participants were Ethiopian nationals, and each study site served populations that are largely linguistically and culturally homogeneous within their respective regions. Although primary language was not systematically recorded at the individual level, routine clinical care and all study interactions (including consent and data collection) were conducted in the dominant local languages at each site (Amharic and Afan Oromo), with facilitators fluent in the language used (Page 5, Line 158-163, Page 16 Line 511-513).

10. (R2): Participant clinical characteristics: The clinical characteristics of child participants presented in Table 1, pages 5 and 6, does not provide information on whether RHD was present (see comment above referring to the use of the term "ARF/RHD" in inclusion criteria). On the assumption that all child participants did have diagnosed RHD, it would be useful to provide further subcategorization of the severity of illness, for example whether their illnesses were symptomatic or asymptomatic, if these data are available. This would help provide further context for interpreting the results, as the lived experience of RHD of those with latent disease would likely differ from those with overt illness.

Thank you for this important suggestion which we have clarified elsewhere and incorporated into the Results and Table 1 (Page 7, Line 225-236).

11. (R2): Discussion, Provider refusal to inject: In Discussion paragraph 2 (page 11) it is indicated that there was "refusal to inject in some centres". According to the results this appeared to be occurring only in one centre. I suggest that this is reworded to "...refusal to inject in one centre..."

We agree and have revised accordingly (Page 15, Line 445).

12. (R2): Supplementary 1 Text: Text Wording of questions/prompts used in focus group discussions. As described in the Methods (paragraph 1, page 5), Supplementary 1 Text provides the guide for facilitating the focus group discussions, and contains the open-ended prompts used in these discussions. The prompt provided for factors impacting prophylaxis delivery services ("What factors influence the delivery of BPG prophylaxis in healthcare settings?") uses health service wording that would not be clear to child participants with a mean age of 13.5 years. It would be helpful to provide the prompt as actually used in the focus group discussions, translated into English.

We thank the reviewer for raising this important point. We agree that the wording presented in Supplementary Text 1 reflects a structured analytic framework used by the research team, rather than the verbatim language used when engaging child participants. In the focus group discussions, facilitators did not use health-service or policy-level terminology (e.g. “structural” or “organizational” factors) with children. Instead, these domains guided the facilitators’ probing internally, while questions posed to children were phrased in simple, age-appropriate language and delivered in the local language. For example, the question listed in the supplement as “What factors influence the delivery of BPG prophylaxis in healthcare settings?” was operationalised in the discussions using child-friendly prompts such as: “Can you tell us about things that make it easy or hard for you to get your penicillin injections?”, with follow-up probes tailored to children’s responses (e.g. experiences at the clinic, interactions with nurses, pain during injections, or family support). To improve clarity and transparency, we have revised Supplementary Text 1.

All authors have approved this resubmission, and we continue to have no conflicts of interest to declare. For any correspondence regarding this manuscript, please contact me at eshetie.birru@thekids.org.au or meshetie21@gmail.com.

Sincerely,

Eshetie Melese Birru, PhD

On behalf of all authors
