# Supplementary material for: Determinants of secondary prophylaxis for childhood rheumatic heart disease in Ethiopia: A qualitative study of children and caregivers
Source: PLoS One. 2026 May 26;21(5):e0349776. doi: 10.1371/journal.pone.0349776 (PMC13210385; doi:10.1371/journal.pone.0349776)
Supplement: Attachment — Submitted filename: Reviewers comments PONED_25_52910R1.docx [file pone.0349776.s006.docx]

This article covers an important global health issue, and a strength is the prioritisation of voices of people affected by rheumatic heart disease. The study extends on a growing body of literature exploring the importance of lived experience in the context of rheumatic fever and rheumatic heart disease.

I do have some comments and recommendations listed below.

Background

On line 71 of the manuscript I suggest you add Indigenous people to those who inequitably experience rheumatic heart disease in medium to high economic countries such as New Zealand and Fiji.

Given that cost is identified as a barrier to receiving secondary prophylaxis in the reported findings, there should be some mention that BPG provision in Ethiopia operates on a fee for service model in the background section of the paper (funding models do vary internationally).

There are now a number of qualitative studies that have explored lived experience of rheumatic fever and rheumatic heart disease – with a focus on children and adolescence. This literature has not been included in the article and could help inform the background contexts and implications of this study. Child-centic research by Julie Spray nicely positions the importance of children’s perspectives, while research by Anneka Anderson, Jane Oliver, Siobhan Tu’akoi and Emma Haynes have all provided insights from Indigenous and Pacific lived experience that align with the findings of this study. I recommend that the paper is strengthened by drawing on literature from similarly based research.

Methods

The article states its focus is on children and aligns with the UNCRC definition of a child being someone under 18 years of age. However, this age range also spans both child and adolescent life stages. Treating this range as a homogenous group can mask age/developmental differences in perceptions of rheumatic heart disease and secondary prophylaxis and in and of itself can generate ageism in blanket assumptions. It would be good to see this explored more in the analysis or at least considered as a limitation.

As the focus groups included children, adolescents, and adult caregivers, how was the power imbalance of adult (and potentially adolescent) voices managed to achieve child voice and perspectives? Similarly, were any child-specific data collection methods applied in the research such as role playing, play-based scenarios, visual or story-based approaches? If these were not included, it would be nice to see a justification of the data collection methods used with a paediatric cohort.

Given that rheumatic fever and rheumatic heart disease are diseases of stigma, there is a risk that focusing on “behaviour drivers” of people with these diseases can re-allocate blame to them and mask the significant impact of broader social determinants of health and health systems, thereby maintaining and perpetuating the stigma. By way of example, the paper attributed poor relationship building as a behaviour of participants. However, relationships are inherently reciprocal, between health providers, communities and families, not just a behavioural ‘fault’ of people living with rheumatic heart disease. The uncritical analysis and framing in this paper for me is problematic. I would recommend that this framing is re-considered with a more structural and strengths-based (non-deficit) interpretation. Alternatively, as the discussion section had more of a focus on structural impacts on secondary prophylaxis adherence than behaviour drivers- I would also consider removing the behavioural focus of the paper.

Results

A key limitation of this paper was the presentation of the findings. There was too little description of the themes and too many quotes, leaving the reader the do all the interpretation. Achieving a balance between description (interpretation) and quotes (evidence) is crucial in qualitative research to provide both authenticity and analytical rigor. The ideal approach is to use quotes to illustrate themes, not to replace interpretation. I recommend that this section is re-written with more emphasis on the descriptions and less use and reliance on the quotes.

Discussion

The discussion could have been strengthened by drawing on similar literature (noted above) and discussing implication of the findings from this study within this broader context.
