# Supplementary material for: Determinants of secondary prophylaxis for childhood rheumatic heart disease in Ethiopia: A qualitative study of children and caregivers
Source: PLoS One. 2026 May 26;21(5):e0349776. doi: 10.1371/journal.pone.0349776 (PMC13210385; doi:10.1371/journal.pone.0349776)
Supplement: Attachment — Submitted filename: Response_to_Reviewers_auresp_2.docx [file pone.0349776.s008.docx]

**Response Letter**

Dear Editor,

Thank you for the opportunity to submit a revised version of the article entitled “Determinants of secondary prophylaxis for childhood rheumatic heart disease in Ethiopia: a qualitative study of children and caregivers.” We are grateful to the reviewers for their constructive comments, which have helped us improve the quality of this work. We have carefully addressed each comment and revised the manuscript accordingly. In particular, we have refined the relevant sections to strengthen the supporting literature, improved methodological transparency, and enhanced the presentation of the findings where suggested.

A detailed, point-by-point response to each comment is provided below, with corresponding revisions clearly indicated in the manuscript.

**Background comments**

**Comment 1**

On line 71 of the manuscript I suggest you add Indigenous people to those who inequitably experience rheumatic heart disease in medium to high economic countries such as New Zealand and Fiji.

***Response:*** *Thank you for this valuable suggestion. We agree that Indigenous populations in high-income settings experience a disproportionate burden of rheumatic heart disease. We have revised the Background to explicitly acknowledge Aboriginal and Torres Strait Islander peoples in Australia, Māori and Pacific peoples in New Zealand, and Indigenous populations in Fiji as populations disproportionately affected by rheumatic heart disease. This revision strengthens the global contextualisation of disease burden (Page 3, Lines 72–73).*

**Comment 2**

Given that cost is identified as a barrier to receiving secondary prophylaxis in the reported findings, there should be some mention that BPG provision in Ethiopia operates on a fee for service model in the background section of the paper (funding models do vary internationally).

**Response:**
*We appreciate this important observation. We have expanded the Background to clarify that BPG provision in Ethiopia occurs within a mixed financing structure, including community-based health insurance and out-of-pocket payments, particularly during medication stockouts. This addition strengthens interpretation of financial barriers reported in the Results and enhances contextual understanding of treatment access (Page 4, Lines 104–109).*

**Comment 3**

There are now a number of qualitative studies that have explored lived experience of rheumatic fever and rheumatic heart disease – with a focus on children and adolescence. This literature has not been included in the article and could help inform the background contexts and implications of this study. Child-centic research by Julie Spray nicely positions the importance of children’s perspectives, while research by Anneka Anderson, Jane Oliver, Siobhan Tu’akoi and Emma Haynes have all provided insights from Indigenous and Pacific lived experience that align with the findings of this study. I recommend that the paper is strengthened by drawing on literature from similarly based research.

**Response:**
*We thank the reviewer for highlighting this important body of literature. We have incorporated relevant qualitative studies that explore child and family lived experiences of rheumatic fever and rheumatic heart disease across Indigenous and Pacific settings. These additions strengthen the Background and Discussion by situating the present findings within existing international qualitative research (Page 4, Lines 111–119).*

**Methods comments**

**Comment 4**

The article states its focus is on children and aligns with the UNCRC definition of a child being someone under 18 years of age. However, this age range also spans both child and adolescent life stages. Treating this range as a homogenous group can mask age/developmental differences in perceptions of rheumatic heart disease and secondary prophylaxis and in and of itself can generate ageism in blanket assumptions. It would be good to see this explored more in the analysis or at least considered as a limitation.

**Response:**
*We appreciate this thoughtful observation. The study population included participants across late childhood and adolescence (mean age 13.5 years), reflecting the epidemiological distribution of rheumatic heart disease. The analytical approach focused on shared caregiving and health system experiences relevant to prophylaxis delivery. However, to address the concern we included this as a limitation of the study (Page 19, Lines 605–609).*

**Comment 5**

As the focus groups included children, adolescents, and adult caregivers, how was the power imbalance of adult (and potentially adolescent) voices managed to achieve child voice and perspectives? Similarly, were any child-specific data collection methods applied in the research such as role playing, play-based scenarios, visual or story-based approaches? If these were not included, it would be nice to see a justification of the data collection methods used with a paediatric cohort.

**Response:**
*Thank you for raising this important methodological consideration. We have expanded the Methods section to clarify that facilitators actively encouraged child participation, used age-appropriate language, and ensured that children were invited to express their views independently. Caregivers were also encouraged to allow children to share their experiences directly. These clarifications strengthen methodological transparency (Page 6, Lines 184–189).*

**Comment 6**

Similarly, were any child-specific data collection methods applied in the research such as role playing, play-based scenarios, visual or story-based approaches? If these were not included, it would be nice to see a justification of the data collection methods used with a paediatric cohort.

**Response:**

*We appreciate the reviewer’s suggestion regarding child-specific qualitative tools. While specialised methods such as visual or play-based techniques were not used, semi-structured focus group discussions were considered appropriate given that most participants were adolescents capable of verbal expression. This approach is consistent with established qualitative methodologies used in paediatric chronic disease research. We have added clarification in the Methods to justify the chosen approach (Page 6, Lines 192–196).*

**Result and Discussion comment**

**Comment 7**

Given that rheumatic fever and rheumatic heart disease are diseases of stigma, there is a risk that focusing on “behaviour drivers” of people with these diseases can re-allocate blame to them and mask the significant impact of broader social determinants of health and health systems, thereby maintaining and perpetuating the stigma. By way of example, the paper attributed poor relationship building as a behaviour of participants. However, relationships are inherently reciprocal, between health providers, communities and families, not just a behavioural ‘fault’ of people living with rheumatic heart disease. The uncritical analysis and framing in this paper for me is problematic. I would recommend that this framing is re-considered with a more structural and strengths-based (non-deficit) interpretation. Alternatively, as the discussion section had more of a focus on structural impacts on secondary prophylaxis adherence than behaviour drivers- I would also consider removing the behavioural focus of the paper.

**Response:**

*We thank the reviewer for this important and thoughtful comment highlighting the risk of deficit-based framing in studies addressing ARF/RHD. We fully agree that ARF and RHD occur within complex social, cultural, and structural contexts, and that attributing challenges in treatment engagement solely to individual behaviours risks reinforcing stigma and obscuring the role of systemic inequities. A growing body of qualitative and lived-experience research has demonstrated that the burden of RHD and engagement with care are strongly shaped by broader social determinants, structural disadvantage, and health-system factors rather than individual deficits alone (Haynes et al., 2020).*

*We respectfully retained the COM-B model as the organising framework because it is an implementation science tool designed to capture influences across individual, relational, and structural domains. Importantly, the COM-B framework conceptualises behaviour as arising from the interaction of capability, opportunity, and motivation, where “opportunity” explicitly includes both physical and social opportunity such as access to health services, availability of resources, environmental conditions, and institutional context (Michie et al., 2011). Therefore, the model does not inherently locate responsibility within individuals but instead enables systematic identification of modifiable determinants across the broader care environment.*

*To address the reviewer’s concern, we have revised the manuscript to clarify this intent and to avoid wording that could be interpreted as deficit-based. Specifically, we replaced language suggesting that relationship-building was a behaviour attributable to participants alone. Instead, trust, communication, and continuity of care are now framed as reciprocal and relational processes shaped by interactions between healthcare providers, families, and health systems. This reframing is consistent with contemporary evidence demonstrating that trust in patient-provider relationships is influenced by communication quality, continuity of care, available time, and organisational context rather than individual behaviours in isolation (Lerch et al., 2024).*

*We also strengthened the Discussion section to more clearly emphasise the structural and organisational determinants identified in this study. These include systemic challenges such as BPG stockouts, transportation burden, financial constraints, provider concerns about severe adverse reactions, fragmented service delivery, and limited counselling opportunities. These factors are well documented contributors to inequitable access to prophylaxis and ongoing care in RHD programs (Wyber et al., 2020).*

*Furthermore, emerging evidence continues to highlight that communication and engagement in healthcare are inherently relational and service-mediated processes. A recent scoping review demonstrated that effective communication between healthcare providers and vulnerable patient populations is strongly associated with improved health outcomes and is influenced by service-level characteristics and communication systems (Jenstad et al., 2024).*

*We also acknowledge the reviewer’s broader concern regarding stigma and deficit discourse. Contemporary lived-experience research in RHD has identified the condition as a marker of profound social inequity and has cautioned against narratives that unintentionally reinforce stigma or institutional bias if structural context is not foregrounded (Haynes et al., 2020). In response, we have revised selected wording throughout the manuscript to adopt a more strengths-based and context-sensitive interpretation of findings.*

*Overall, these revisions preserve the practical utility of the COM-B framework for translating lived experiences into actionable implementation domains while ensuring that responsibility is not inappropriately attributed to children or caregivers. Instead, determinants are more explicitly framed as shared, relational, and system-influenced processes that require coordinated health-system responses (Areri et al. Barriers and facilitators of benzathine penicillin G adherence among rheumatic heart disease patients: a mixed methods systematic review using the COM-B (capability, opportunity, and motivation for behavior) model. Syst Rev. 2024 Dec 3;13(1):297. doi: 10.1186/s13643-024-02691-1).*

**Comment 8**

The discussion could have been strengthened by drawing on similar literature (noted above) and discussing implication of the findings from this study within this broader context.

**Response:**
Thank you for this helpful recommendation. We have strengthened the Discussion by incorporating additional qualitative literature from Indigenous and Pacific settings and comparing our findings with similar structural and experiential determinants identified in previous studies. This revision strengthens interpretation and situates our findings within the broader global context of rheumatic heart disease care (Page 17, Line 510-522).

On behalf of all authors

Eshetie Melese Birru
